# Colloidal Synthesis of Bulk-Bandgap Lead Selenide Nanocrystals

**DOI:** 10.3389/fchem.2018.00562

**Published:** 2018-11-22

**Authors:** Thulitha M. Abeywickrama, Asra Hassan, Preston T. Snee

**Affiliations:** Department of Chemistry, University of Illinois at Chicago, Chicago, IL, United States

**Keywords:** lead selenide, quantum dots, semiconductor, nanomaterials, conductivity

## Abstract

Lead selenide quantum dots (QDs) are low-bandgap IV-VI semiconducting nanomaterials that have been studied for a variety of applications. Their preparation using colloidal methods can create small spherical to larger cubic nanocrystals, with an upper limit of ~17 nm reported to date. Here we describe methods for preparing cubic PbSe nanocrystals over a 20–40 nm size range using a twostep procedure. Specifically, ~10 nm PbSe QDs are generated using the rapid injection method, the products from which are overcoated with additional lead and selenium precursors. The use of two lead reagents were studied; lead oleate resulted in a maximum of 20 nm cubes, while more reactive lead hexyldecanoate resulted in much larger nanomaterials with bulk bandgaps. However, PbSe samples prepared with lead hexyldecanoate also contained agglomerates. Special care must be taken when characterizing larger strained nanomaterials with X-ray powder diffraction, for which the Scherrer equation is inadequate. A more rigorous approach using the Williamson–Hall method provides characterizations that are consistent with electron microscopy analysis.

## Introduction

Quantum dots (QDs) are nanoscale particles that have size-tunable optoelectronic properties that may differ substantially from their bulk counterparts. The materials can be prepared using low-cost colloidal methods, which has attracted attention for their use in electronic devices (Talapin and Murray, 2005; Talapin et al., 2010), photovoltaics (Tang et al., 2011; Etgar et al., 2013), sensors (Snee et al., 2006), and in bioimaging (Alivisatos, 2004). Quantum dots are often classified based on the elemental grouping of their composition, which in turn is associated with specific applications. For example, II–VI's such as CdSe are visible emitters often employed in biological sensing applications and have been incorporated into commercially available displays (i.e., television sets). The III–V's such as InP and InAs are of interest for their near-IR emission, and the IV–VI's such as PbS and PbSe are low bandgap materials that have utility in electronics, photodetectors, and solar cells. Lead selenide has been extensively studied due to the high quality of the nanomaterials that can be prepared (Pietryga et al., 2004; Capek et al., 2015) and the ease of tunability of the electronic structure due to the low bulk bandgap (0.27 eV) and large excitonic Bohr radius (46 nm) (Yanover et al., 2012). There are many procedures to synthesize PbSe QDs, although the most robust utilize the rapid injection method since it can produce QDs with narrow, near-atomically precise size distributions (Capek et al., 2015; Shapiro et al., 2016).

Previously reported methods to prepare PbSe QDs often employ high boiling point organic solvents (diphenyl ether or 1-octadecene) containing stabilizers such as oleic acid, into which lead salt and phosphine selenide precursors are injected. For many years the reaction yields were reported to be very low, which was found to result from the near ubiquitous use of trioctylphospine selenide (TOPSe) as the calchogenide precursor (Evans et al., 2010). Specifically, Evans et al. discovered that purified TOPSe is non-reactive with lead and that previous reports on preparing PbSe QDs using this reagent relied on the fortuitous presence of secondary phosphine impurities (Evans et al., 2010). As such, it is common to incorporate diphenylphosphine with TOPSe in a preparation. At present, there are reports of the colloidal synthesis of small magic-sized clusters to larger PbSe cubes on the order of 15–17 nm, which imparts bandgaps from the higher energy near infrared to shortwave infrared spectral regions (Shapiro et al., 2016).

There is interest in the synthesis of larger nanomaterials using colloidal techniques. For example, Zamkov and co-workers prepared very large (~25 nm) nanocrystals by ligand-induced digestive ripening of as-prepared CdSe and CsPbBr_3_ QDs among others (Razgoniaeva et al., 2018). Nanomaterials of this size have minimal interfacial areas, which enhances the conductivity of films made from these systems (Razgoniaeva et al., 2017). We sought to prepare very large monodisperse PbSe nanocrystals for these reasons and to challenge the present ~15–17 nm size limit (Pietryga et al., 2004; Shapiro et al., 2016). To this end, we explored the use of lead acid precursors of varying reactivity, which resulted in the development of two protocols to produce cubic PbSe QDs over a 20–40 nm in size range. This was achieved by a two-step process whereby ~10 nm core QDs were overcoated to obtain much larger cubic QDs. The main factors that dictate the sizes and quality of the products are the growth temperature and the identity of the lead precursor. Special care must be taken when characterizing larger strained crystalline nanomaterials using powder X-ray diffraction techniques, for which the Scherrer equation is inadequate. Last, it was found that the larger sized PbSe samples are significantly more conductive than their smaller counterparts.

## Experimental

### Materials

Acetone (99.5%), diphenyl phosphine (98%), hexane (mixture of isomers, 98.5%), 2-hexyldecanoic acid (96%), lead(II) oxide (PbO, 99%), methanol (99.8%), 1-octadecene (technical grade, 90%), oleic acid (90%), oleylamine (>98% primary amine), 2-propanol (99.5%), tetrabutylammonium iodide (98%), triethylamine (99%), and trifluoroacetic anhydride (99%) were purchased from Sigma-Aldrich. Acetonitrile (HPLC Grade) and trifluoroacetic acid were purchased from Fisher Scientific. Trioctylphosphine (97%) was purchased from Strem. Trioctylphosphine selenide (TOPSe) were prepared by mixing Se metal into trioctylphoshpine and stirring overnight to prepare a 1M solution. Diphenylphosphine selenide was prepared according to the procedure of Evans et al. (2010). Oleic acid was recrystallized according to the procedure of Arudi et al. (1983).

#### Preparation of Pb(C_18_H_33_O_2_)_2_

Lead oleate was synthesized using lead oxide, trifluoroacetic anhydride, and oleic acid as described by Hendricks et al. (2015).

#### Preparation of Pb(C_16_H_31_O_2_)_2_

Lead hexyldecanoate was synthesized using a modification of the method reported by Hendricks et al. (2015). Lead(II) oxide (5.000 g, 22.4 mmol) and 10 mL acetonitrile were added to a 100 mL round bottom flask maintained in an ice bath. After stirring for 10 min, trifluoroacetic acid (0.34 mL, 4.48 mmol) and trifluoroacetic anhydride (3.20 mL, 22.4 mmol) were added. The solution turned clear after 15 min stirring and was then warmed to room temperature. In another 250 mL round bottom flask, 2-hexyldecanoic acid (12.2 mL, 45.0 mmol) and triethylamine (7.13 mL, 50.5 mmol) were mixed in 90 mL of 2-propanol. The lead trifluoroacetate solution was then added to the 2-hexyldecanoic acid solution. The resulting colorless mixture was heated to reflux for 2 h and then cooled to room temperature. The solution was concentrated under vacuum and the precursor was separated as a semisolid by filtration after cooling to the point where solid materials separated. To remove excess triethylamine, the crude product was heated with an attached distillation apparatus with a temperature probe until a condensate appeared at 89°C, which is the boiling point of triethylamine. The product was left at this condition for ~1 h to assure the removal of the amine and was then characterized with NMR (Figure S1) and combustion analysis (Table S1). Deviations from the expected results are due to residual triethylamine; these data are provided in the supporting information.

#### Preparation of “core” PbSe QDs

PbSe QDs were synthesized using the hot injection method via two protocols that are derivatives of that reported by Shapiro et al. (2016).

#### Method (1) stoichiometric ligand condition

Into a 50 mL three neck round bottom flask containing 7.5 g 1-octadecene (ODE) solvent, either 0.483 g lead oleate (0.63 mmol) or 0.450 g lead hexyldecanoate (0.63 mmol) was added. The solution was heated under vacuum at 100°C until the pressure stabilized. The selenium precursor in the injection solution was prepared inside a glovebox by mixing 0.4 g of 1.0 M trioctylphosphine selenide (TOPSe, 0.48 mmol) with 0.15 g of diphenylphosphine (DPP, 0.81 mmol) with 1.2 g of trioctylphosphine (TOP), which was then loaded into a 3 mL syringe. The lead precursor/ODE solution was heated to 260°C under nitrogen, at which point the heating mantle was removed. The selenium solution was rapidly injected when the solution cooled to 240°C. This solution was maintained at 230°C for another 10 min before cooling to room temperature.

#### Method (2) excess ligand condition

Another set of PbSe QDs were prepared following the same procedure above with excess ligand present in the lead precursor/ODE solution. Specifically, 0.551 g of oleic acid (1.95 mmol) was added to the 50 mL three neck round bottom flask when using lead oleate as a precursor. Alternatively, 0.500 g of 2-hexyldecanoic acid (1.95 mmol) was added when using lead hexyldecanoate. The preparation of magic-sized PbSe QDs was attempted using lead hexyldecanoate as above, with the exception that the solution was maintained at room temperature for a 24 h growth period.

An XPS survey spectrum for the processed lead oleate “core” QDs was measured (Figure S2). The elemental composition from the XPS analysis is presented in Table S2. These data reveal the presence of lead and selenium as well as some oxide. The Pb:Se ratio is 2:1, which is a common observation from PbSe QDs and likely results from the presence of excess Pb precursors that were not removed upon precipitation/washing of the sample before analysis (Jawaid et al., 2011).

### Overcoating of PbSe QDs

Due to the low quality of “core” PbSe nanomaterials prepared with lead hexyldecanoate, all subsequent manipulations were performed with PbSe QDs from Method 2 with the lead oleate precursor. This method produced the largest, most homogeneous QDs. When overcoating a sample, a portion (typically ½) of a batch of the as-prepared PbSe QDs were transferred into a 20 mL vial. Addition of hexane and increasing amounts of 2-propanol resulted in precipitation, and the QDs were separated by centrifugation. The precipitate was re-dissolved in a hexane/2-propanol mixture and centrifuged again; this step was repeated twice more.

#### Method (1) overcoating with Pb(C_18_H_33_O_2_)_2_

Approximately half of a batch of as-prepared “core” QDs were processed as above. The precipitated nanocrystals were added to 20 g ODE containing 0.121 g oleic acid (0.43 mmol) in a 100 mL four neck round bottom flask. The mixture was degassed under the vacuum at 100°C until the pressure stabilized. Then the solution was heated to either 130, 160, or 190°C under a nitrogen atmosphere. Meanwhile, two precursor solutions were prepared for injection. One for lead was prepared by dissolving 0.290 g of lead oleate (0.38 mmol) in 3 mL TOP, and the selenium solution was prepared by dissolving 0.100 g diphenylphosphine selenide (DPPSe, 0.38 mmol) in 3 mL TOP. These manipulations were performed in a glovebox. Once the QD/oleic acid/ODE bath reached the target temperature, the lead and selenium precursor solutions were added using a syringe injector at a 3 mL/h rate while maintaining a constant temperature. After complete addition of the precursors, the mixture was maintained at the overcoating temperature for another 5 m before cooling to ambient. Products were stored as-prepared in a glove box until later analysis.

Two additional protocols were investigated to prepare larger materials. Both methods sought to increase the precursor to QD ratio in the overcoating process. One was realized by doubling the precursors' masses; specifically, 0.580 g of lead oleate was (0.75 mmol) was added to 3 mL TOP and 0.200 g of DPPSe was likewise added to 3 mL TOP, which were subsequently injected into a QD/oleic acid/ODE solution at 160°C. Another method used the original masses of lead and selenium precursors, which were applied to ~¼ of a batch of “core” PbSe QDs.

#### Method (2) overcoating with Pb(C_16_H_31_O_2_)_2_

This procedure is similar to Method 1 except where noted. Approximately half of a batch of “core” QDs prepared via Method 2 using lead oleate were processed using hexane/2-propanol as discussed previously. The solvent consisted of a solution of 0.110 g 2-hexyldecanoic acid (0.43 mmol) in 20 g ODE. The injection solutions consisted of 0.540 g lead hexyldecanoate (0.75 mmol) in 3 mL TOP and 0.200 g DPPSe (0.75 mmol) in 3 mL TOP. The overcoatings were performed at either 130 or 160°C. Products were stored as-prepared in a glove box until later analysis.

### Characterization

Samples were characterized by removing an appropriately-sized portion from storage in the glovebox, which was centrifuged to separate the PbSe precipitate. The solid was re-dissolved in hexane and centrifuged again; this step was repeated once more. The solid material was re-dispersed in hexane or tetrachloroethylene and was used for further characterizations. Transmission Electron Microscopy (TEM) analyses were performed using JEOL JEM-3010 microscope operating at 300 KeV. TEM grids were prepared by dropcasting a drop of the processed nanoparticle solution onto a 300 mesh carbon-coated Cu grid from Ted Pella. X-ray diffraction measurements were performed using a D8 Advance ECO Bruker XRD diffractometer with Cu Kα (λ = 1.54056 Å) radiation. The instrument was used in both a low and high-resolution modes. The angle-dependent instrument response function of the XRD diffractometer was determined using NIST SRM 640e, a strain-free Si standard composed of ~4.1 micron sized particles. X-ray photoelectron spectroscopy (XPS) analysis was performed on a Kratos Axis 165 with Al Kα source operate at 12 kV and 10 mA. Fourier-transform infrared spectroscopy analysis was performed using an Thermo Nicolet 6700 FTIR and a Bruker Tensor 27 FTIR. Samples for FTIR analyses were coated onto a KBr pellet. ^1^H NMR and ^13^C NMR spectra were recorded using a Bruker Avance DRX 400 NMR spectrometer, and elemental analysis was performed by Midwest Microlab.

Conductivity measurements were performed on “core” ~12 nm PbSe QDs prepared with method 2 and larger ~22 nm PbSe overcoated samples. The processing is based on the method of Ning et al. (2014), whereby 1 mL of growth solution was washed with anhydrous hexane and centrifuged 3 × . The samples were redispersed into hexane and incubated with 0.6 mL of a 0.6 M solution of tetrabutylammonium iodide in oleylamine for 15 min in a glove box. The samples were washed with excess dry methanol and then hexane, and were redispersed in 2 mL hexane with sonication before spin coating onto a lithographically prepared Si substrate consisting of two Ti electrodes with a 3 μm gap. The samples were dried under vacuum, and then taken out into ambient air and light for measuring current vs. applied voltage using a Kiethley 2450 Sourcemeter, with the voltage swept from −3.00 to +3.00 V in steps of 0.1 V. Five devices were measured and were all found to be qualitatively similar, with differences attributed to lithographic imperfections and the presence of residual photoresist in the device's electrode gap.

## Results and discussion

Large 20–40 nm cubic-shaped PbSe colloidal nanocrystals were synthesized from “core” PbSe QDs that were overcoated with additional lead and selenium precursors. The first consideration toward the development of protocols was given to precursors. For the chalcogenide, there has been considerable effort to study the chemistry of selenium since the report by Evans et al. whom demonstrated the necessity of a secondary phosphine species in the production of lead selenide QDs (Evans et al., 2010). As a result, many groups have employed TOPSe with additional secondary diphenylphosphine to prepare PbSe QDs. We have used the same system for selenium, and as such we sought to study more effective lead-carboxylate precursors to realize larger nanomaterials. In this regard, recently Peng and co-workers reported the use of “entropic” carboxylate fatty acid ligands for quantum dot core and core/shell synthesis (Yang et al., 2016). In their vocabulary, an entropic ligand includes 2-hexyldecanoic acid, which is a non-viscous liquid at room temperature despite its very high molecular weight. The Peng group reported that this ligand enhances the solubility of large QDs, and that the extra solubility aids in the overcoating of CdSe QDs into larger CdSe/CdS core/shell sizes (Zhou et al., 2017; Lai et al., 2018). As a result, we examined the use of lead oleate as well as “entropic” lead hexyldecanoate as precursors for PbSe QDs synthesis and subsequent overcoating. Several other parameters such as temperature and stoichiometries were also investigated.

### Synthesis of “core” PbSe QDs

**Figure 1:**
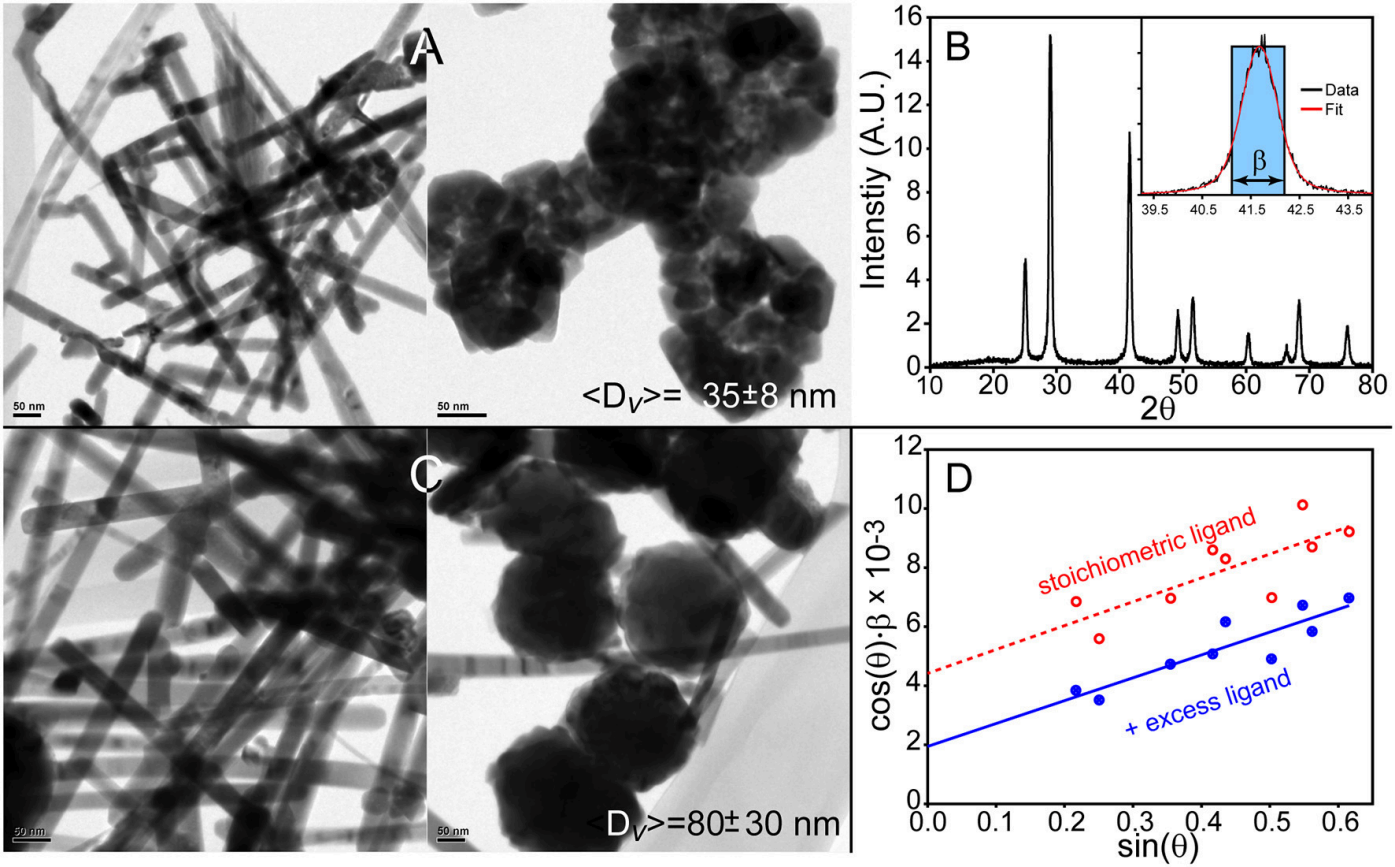
Synthesis of PbSe nanostructures using lead hexyldecanoate results in lead selenide rods and agglomerates of materials. **(A)** Method 1 produces~20 nm thick PbSe rods and agglomerated materials. **(B)** Powder X-ray diffraction pattern of the materials shown in **(A)**. Inset is an example of a resonance's integral breadth. **(C)** Nanostructures prepared with additional hexyldecanoic acid in the solvent results in larger materials although the overall morphology is poorly defined. **(D)** The Williamson–Hall method was applied to the XRD patterns of the materials produced by these methods to determine the volume-weighted average size 〈*D*〉_*v*_.

The synthesis of PbSe QDs was studied by injecting a solution of TOPSe and DPP in TOP into a hot ODE solvent containing either lead oleate or lead hexyldecanoate. Concerning the latter, our hypothesis is that we could create the largest monodisperse PbSe dots or cubes with hexyldecanoic acid due to enhanced solubility of PbSe particles coated with this “entropic” ligand. However, since we must incorporate the ligand as part of the lead precursor to assure a consistent coating of the QDs, it is necessary to use lead hexyldecanoate as the Pb source. It was found that this precursor is highly reactive as its use resulted in the formation of rod-like structures with ~20 ± 5 nm diameters and large quantities of bulky, aggregated nanoparticles as seen in Figure 1A. Powder X-ray diffraction (XRD) characterization (Figures 1B,D) was able to provide a measure of the volume-weighted average size of the ensemble 〈*D*〉_*v*_, which is 35 ± 8 nm. Note that the reported error reflects the systematic uncertainty in the measurement and not necessarily the width of the size distribution; more discussion on XRD analysis is provided below. Inclusion of extra ligands in the solvent resulted in thicker rods as seen in Figure 1C (37 ± 9 nm, as measured by TEM) and more agglomeration as revealed by the increase in the ensemble average 〈*D*〉_*v*_ = 80 ± 30 nm as measured by XRD, see Figure 1D. We believe that the high reactivity of lead hexyldecanoate under these conditions leads to a rapid and rather uncontrolled growth of PbSe nanocrystals no matter our attempts to modify the reaction conditions. As such, it was determined that lead hexyldecanoate is not a good reagent for preparing “core” PbSe QDs using the rapid injection method.

**Figure 2:**
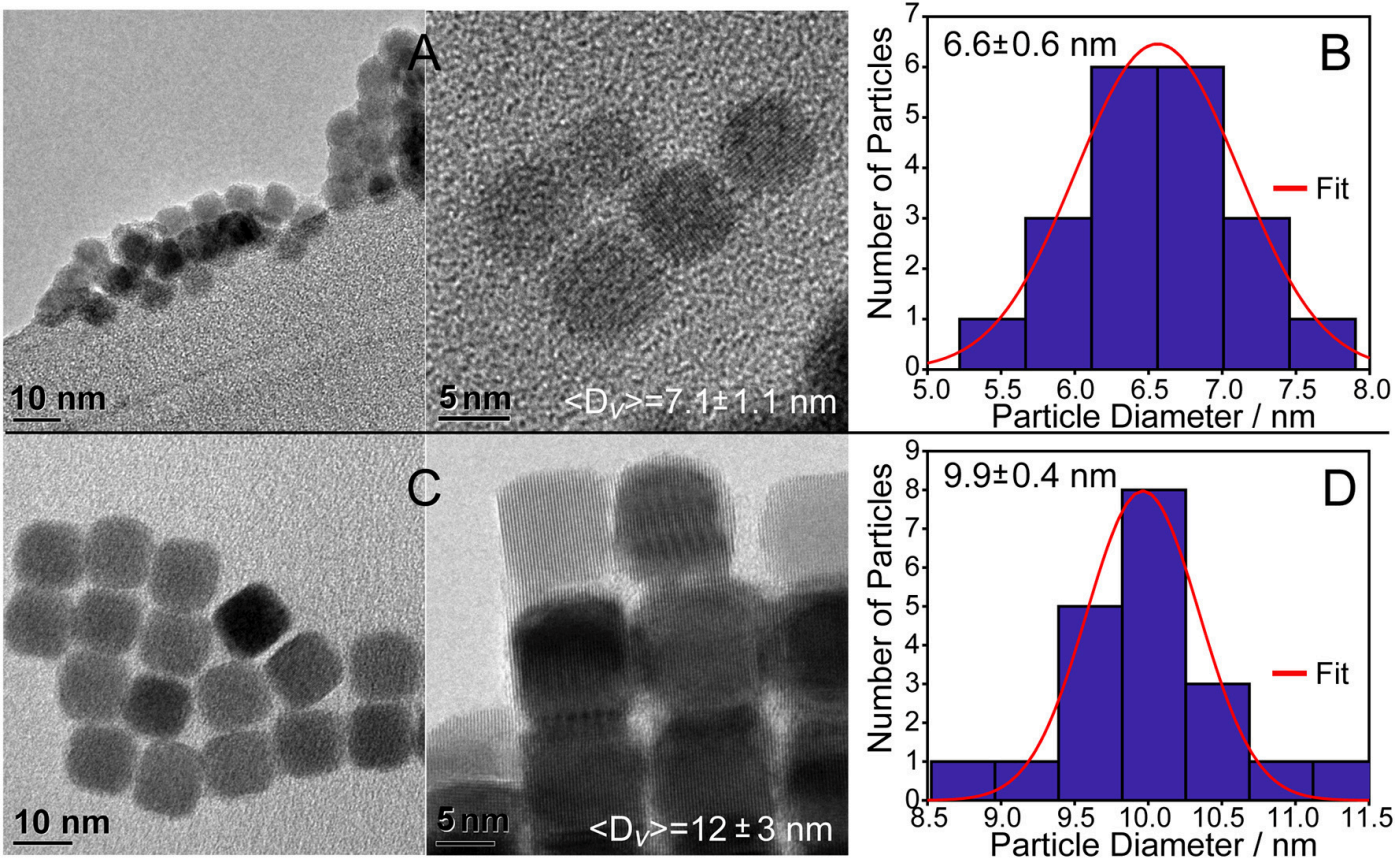
PbSe cubes synthesized using lead(II) oleate. **(A)** Using the reagent without additional oleic acid results in 7.1 ± 1.1 nm cubes as determined using the Williamson–Hall method. **(B)** Histogram of the cube sizes by TEM is consistent with the Williamson–Hall result from XRD analysis. **(C)** Preparing PbSe cubes with additional oleic acid in the solvent results in larger, more homogeneous materials. Additional images are provided in the supporting information, Figure S3. **(D)** Histogram of the cube facet sizes of materials shown in C by TEM is consistent with the results from XRD analysis.

Monodisperse PbSe spheres and cubes were realized using lead oleate with and without additional oleic acid in the solvent as seen in Figures 2A,C. TEM micrographs of the QDs formed under the stoichiometric ligand condition (Method 1) show spheres and some cube-shaped particles with a size distribution of 6.6 ± 0.6 nm (Figure 2B), which is consistent with the XRD measurement of 〈*D*〉_*v*_ = 7.1 ± 1.1 nm. QDs formed under extra ligand conditions are cubic-shaped particles with a size distribution of 9.9 ± 0.4 nm (Figure 2D), which again correlates with the ensemble average of 〈*D*〉_*v*_ = 12 ± 3 nm. Other characterization data such as XPS and elemental analyses as well as additional TEM images (Figure S3) are presented in the supporting information.

### Overcoating of “core” PbSe QDs

**Figure 3:**
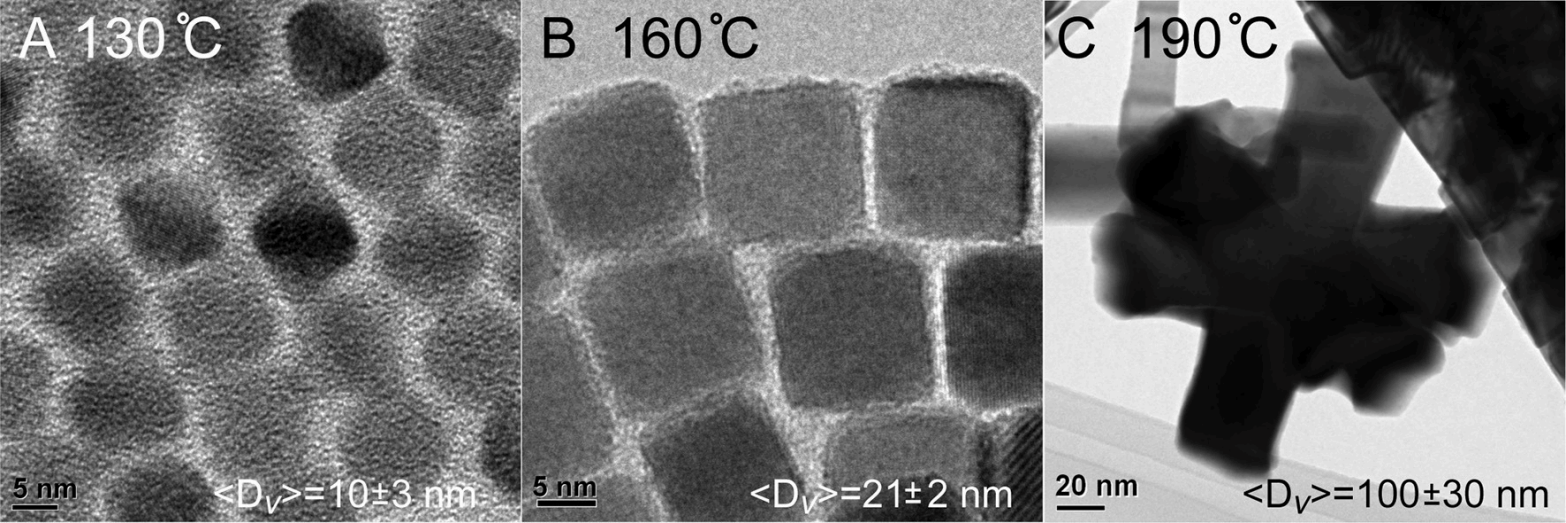
Material size vs. temperature during the overcoating process. **(A)** PbSe cubes do not experience an increase in size when overcoating at 130°C. **(B)** Higher temperatures result in PbSe cubes with~8 × more volume than the starting core material. **(C)** Increasing the temperature to 190°C results in uncontrolled growth.

QDs synthesized with the lead oleate precursor under extra ligand conditions (Method 2) were used as seeds for the addition of a PbSe coating to create larger nanocrystals. In overcoating Method 1, these materials were augmented by the slow addition of lead oleate and highly reactive diphenylphosphine selenide. The overcoating was performed as a function of temperature, with trials at 130, 160, and 190°C. The TEM images of the resulted nanostructures are presented in Figure 3. The particles prepared at the lowest temperature of 130°C (Figure 3A) did not show any detectable growth as 〈*D*〉_*v*_ = 10 ± 3 nm; clearly the temperature was insufficient to activate one or more of the precursors. The best overcoating was realized at 160°C (Figure 3B), which formed the most monodisperse, largest cubic QDs with an ensemble average size of 〈*D*〉_*v*_ = 21 ± 2 nm. At the highest temperature of 190°C the addition of overcoating precursors resulted in large agglomerated structures formed by the aggregation of smaller particles (Figure 3C), which is consistent with the ensemble average of 〈*D*〉_*v*_ = 100 ± 30 nm. Additional TEM images of the nanocrystals formed under these conditions are shown in the supporting information (Figures S4, S5).

**Figure 4:**
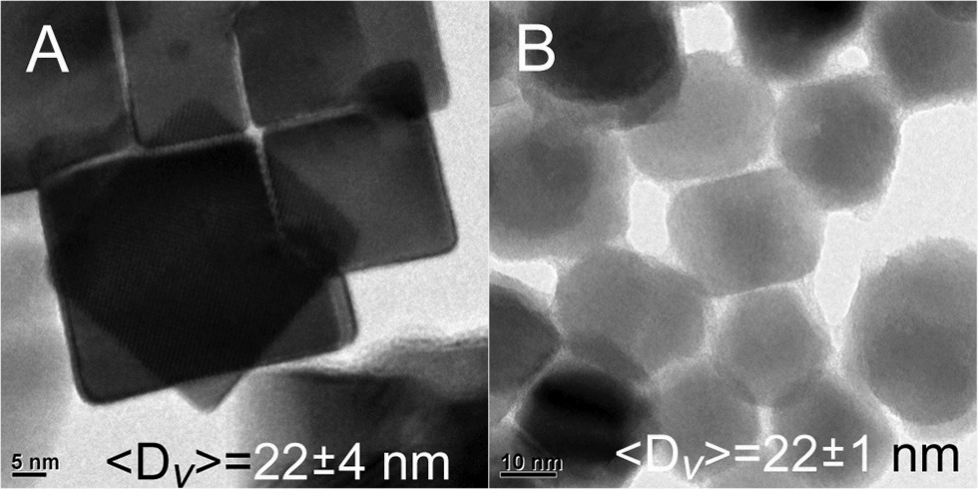
**(A)** Doubling the amount of Pb and Se precursors in the overcoating process does not change the size of the resulting nanomaterials. **(B)** No significant increase in the sizes of materials is realized by overcoating less~10 nm PbSe cubes, although a loss of the cubic morphology is observed.

To create even larger materials, the quantity of overcoating precursors was raised 2 × . Unfortunately, the average ensemble size was not significantly increased (〈*D*〉_*v*_ = 22 ± 4 nm), and the materials appear unaltered as shown in Figure 4A. More TEM images of these structures are shown in the supporting information (Figure S6). Another attempt to push the limits of size was pursued by lowering the quantity of core QDs by ½ and repeating the overcoating exactly as before at 160°C. Again, there was no significant increase in size (〈*D*〉_*v*_ = 22 ± 1 nm), although it was noted that there was some alteration of the sample into a more spherical shape as observed in Figure 4B.

**Figure 5:**
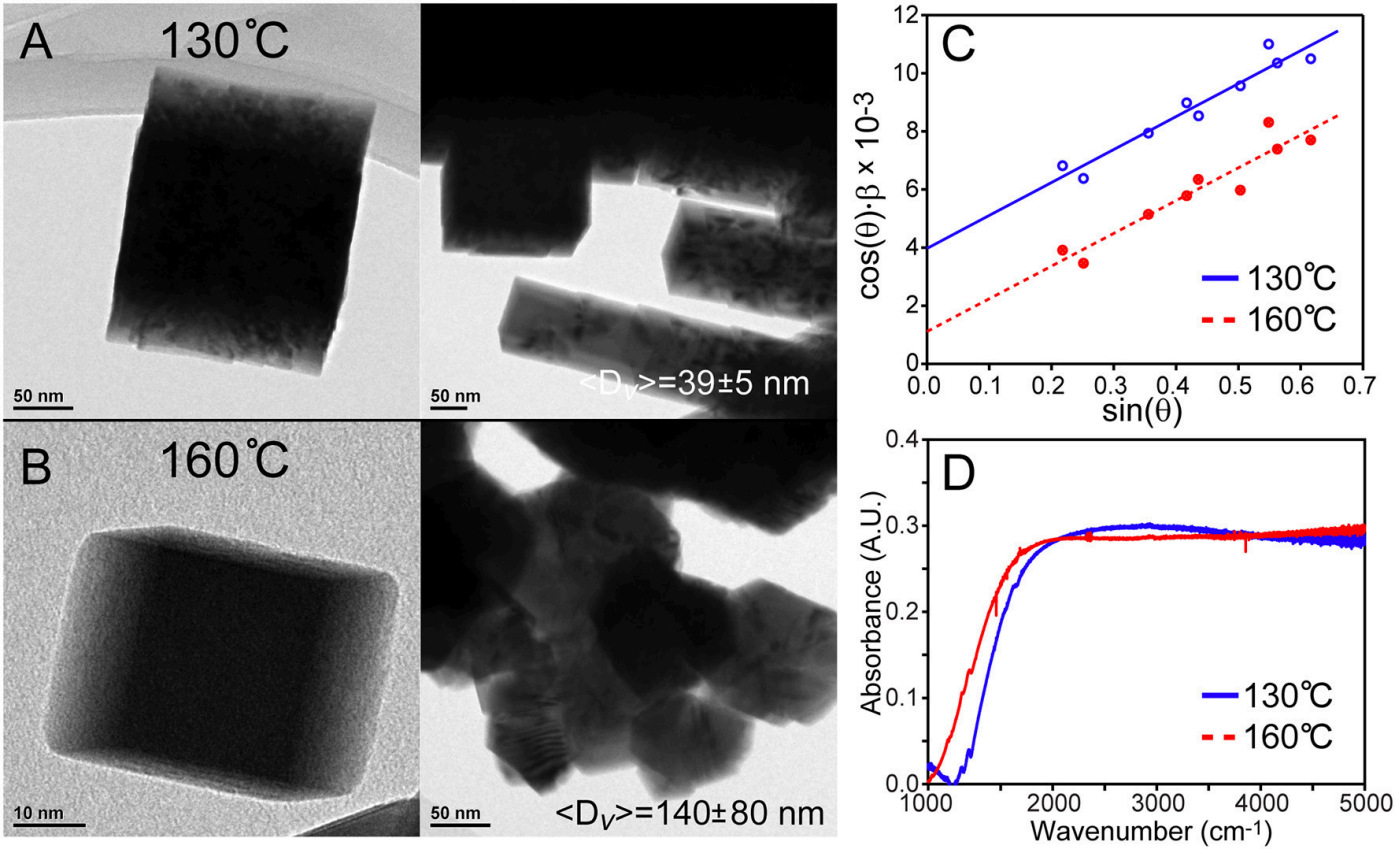
Overcoating PbSe cubes with lead hexyldecanoate. **(A)** Overcoating at 130°C results in singular large cubic materials with 〈*D*〉_*v*_ = 39 ± 5 nm as well as agglomeration. **(B)** Overcoating at a higher temperature creates larger materials with more agglomeration. **(C)** Williamson–Hall analyses reveal strain in these large overcoated PbSe nanostructures due to the finite slopes. **(D)** FTIR spectra of the materials demonstrate bulk bandgaps. Surface ligand features are not observed in larger PbSe nanomaterials that have been washed with hexane thoroughly; note that the contribution from atmospheric species have been subtracted for clarity. The structure of the absorption features is not consistent with the expected rise in the density of states of a bulk semiconductor, and may be due to an artifact of unknown origin.

The use of lead hexyldecanoate was examined in the overcoating process, because although it is too reactive to generate monodisperse “core” PbSe QDs via rapid injection, this same property may engender efficacy when used at lower concentrations and temperatures. The results were successful in part; using this precursor for overcoating at 130°C resulted in the formation of some stand-alone very large, cubic shaped particles (〈*D*〉_*v*_ = 39 ± 5 nm) along with stacked, rod-like shaped agglomerates as seen in Figure 5A. Raising the temperature to 160°C resulted in the formation of bulkier structures (〈*D*〉_*v*_ = 140 ± 80 nm), cubic rods, and a few stand-alone particles as shown in Figure 5B. Additional TEM images are provided in Figures S7, S8 of the supporting information. These results present a mixed message; for one, it is possible to prepare colloidal PbSe nanomaterials >20 nm by increasing the reactivity of the precursors. However, lead hexyldecanoate is too reactive to be used for preparing cores using the rapid injection technique or to overcoat materials due to the loss of monodispersity in the products. To find some use of this reagent, we examined the room temperature synthesis of PbSe QDs rather than smaller magic-sized particles (<2 nm) as observed by Evans et al. (2008). In fact, it was found that ~4.8 nm PbSe QDs were produced simply by mixing lead hexyldecanoate with the selenium source in ODE overnight.

An attempt was made to optically characterize quantum confinement effects on these large PbSe nanocrystals using FTIR as shown in Figure 5D. These spectra demonstrate bandgaps (~0.15 eV) that are lower than bulk PbSe (0.27 eV). This may be due to doping of the dots by excess lead or ambient oxygen, or defects that create states that tail into the bandgap. Significant difficulty was encountered with characterizing smaller QDs, which may be due to the formation of plasmonic states or simple scattering of light from a QD film on a KBr pellet. These effects are currently being investigated, as well as the development of a robust method for optical characterization.

### Powder X-ray diffraction characterization

The application of powder X-ray diffraction analysis to large nanomaterials requires greater attention compared to smaller QDs. This is because the lineshapes of X-ray diffraction resonances are sensitive to size, strain, and instrumental broadening (Balzar, 1999). It is well-known that the Scherrer Equation, ${\langle D\rangle }_{v}=\frac{K\cdot \lambda }{\beta \left(2\theta \right)\cdot cos\left(\theta \right)}$, relates the volume-weighted average size of a particle 〈*D*〉_*v*_ to the 2θ full-width-half-maximum β(2θ) of a resonance. The size also depends on the wavelength of the diffractometer λ, the θ angle of the resonance, and a shape factor K that describes the particles under study. The β factor can also be calculated using the transition's integral breadth, which is the width of a rectangle with the same peak intensity and area as the resonance; see the inset of Figure 1B. The use of this parameter negates the need for a shape factor and can be incorporated into more rigorous XRD characterization techniques such as the Williamson–Hall analysis discussed below (Williamson and Hall, 1953).

We have spent considerable time studying the samples using XRD and found that the need for enhanced analysis is dependent on the particle size. It was found that, under the conditions that the analyses were conducted, the instrumental broadening of resonances has a minor effect on the calculation of the sizes of small particles. For example, ignoring instrumental lineshape broadening results in a 9% underestimation of the size of ~12 nm particles; see Figure S9A of the supporting information. However, greater errors are realized when analyzing larger samples, and the instrument broadening must be characterized. To do so, a NIST standard was measured under the same conditions as the sample, the resonances of which were used as the angle-dependent instrument response function. With these data, the effect of instrument broadening can be removed either by Fourier deconvolution, convolution of a fit with the instrument response function, or by simple subtraction of the standard's integral breadth from the same of the sample. All of these approaches have unique strengths and weaknesses, and require adjustment of the instrument response as a function of diffraction angle. To illustrate the need for instrument response deconvolution, ignoring the instrumental broadening in the case of a nominally 80 ± 30 nm sample results in an underestimated size by 44% as shown in Figure S9B.

Next, the potential for strain broadening of the XRD lineshapes has to be ascertained. For smaller particles, the Scherrer equation is adequate for characterizing QDs so long as they are ~10 nm or smaller and strain free. This was not found to be the case for larger PbSe particles, and they must be analyzed using the Williamson–Hall equation:

(1) $$\begin{array}{l} \beta \left(2\theta \right)\cdot cos\left(\theta \right)=\frac{\lambda }{{\langle D\rangle }_{v}}+4\cdot \epsilon \cdot sin\left(\theta \right) \end{array}$$

where ϵ is the strain that can be crystallographic direction-dependent. Thus, a plot of the instrument response-corrected integral breadth × cos(θ) vs. sin(θ) yields a straight line with an intercept that provides the volume weighted average size of the particles. It was found that the use of Williamson–Hall equation is necessary for characterizing larger PbSe nanomaterial sizes with XRD data. Several examples can be seen in Figures 1D, 5C, as well as Figure S9 of the supporting information. All the Williamson–Hall linear fits have positive slopes, which is indicative of strain. The fact that the samples' XRD patterns have strain broadening reveals that use of the Scherrer equation would result in an underestimation of the particle sizes. Furthermore, the analysis allows us to determine that the larger PbSe materials produced during overcoating consistently have more strain than the smaller “core” PbSe QDs. This indicates that the growth of the shell proceeds with some defect formation. In the future we will examine these materials using more sophisticated techniques such as the Warren–Averbach (Warren and Averbach, 1952) or Rietveld analysis (Rietveld, 1969) techniques. While powerful, these methods require very high quality XRD data with superior knowledge of the angle-dependent instrument response function.

### Conductivity

**Figure 6:**
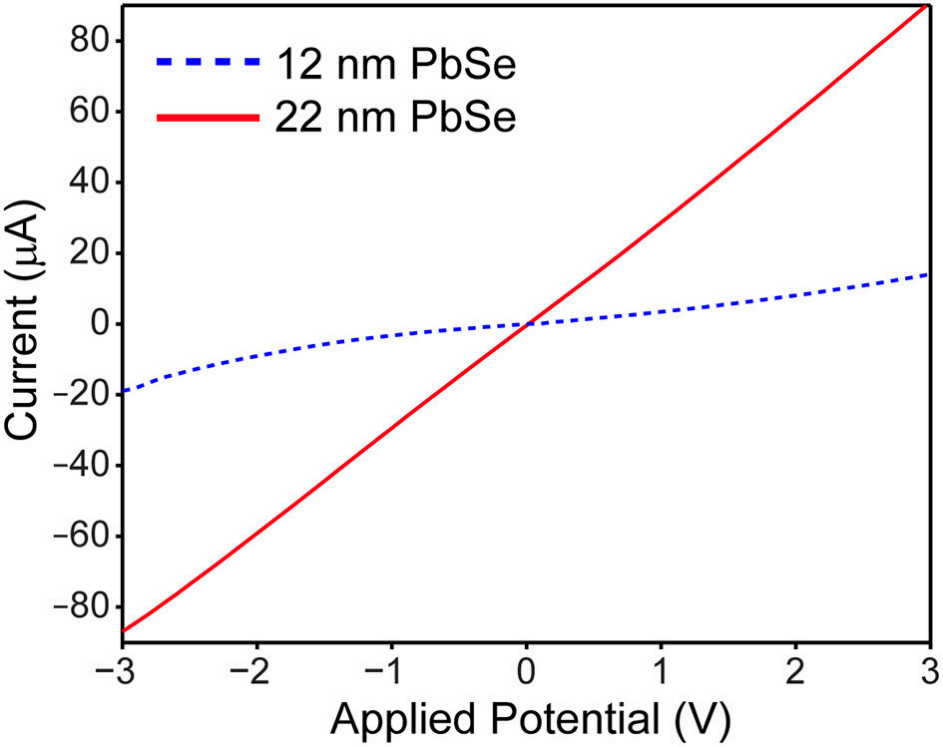
Current vs. voltage plot of ~12 nm PbSe (dotted blue line) vs. ~22 nm PbSe (solid red line) quantum dots reveals that the larger nanomaterials are more linear, symmetric, and ~10 × more conductive than their smaller counterparts.

To verify the enhanced electrical properties of larger PbSe nanomaterials, the conductivity of small 12 nm vs. larger 22 nm samples were measured across a 3 μm Ti gap under ambient conditions. The quantum dots were washed with n-doping tetrabutylammonium iodine solution prior to measurement, to both enhance the conduction and provide protection from ambient oxygen (Ning et al., 2014). As shown in Figure 6, the I-V_bias_ curve is linear, symmetric, and greater in magnitude for the 22 nm PbSe nanomaterials compared to the smaller. These results are consistent with previous studies as electrons and holes must experience less activation energy to make ultimately fewer hops between adjacent ~22 nm quantum dots (Romero and Drndic, 2005; Mentzel et al., 2008; Liu et al., 2010). The asymmetric and non-linear I–V_bias_ curve observed for the smaller QD sample may result from diode-like behavior from Schottky barriers due to the use of low workfunction Ti electrodes (Weiss et al., 2008; Strasfeld et al., 2012); these issues will be the subject of further investigation.

## Conclusion

Presented here is a core/shell growth protocol for producing 20–40 nm PbSe cubic nanomaterials. These large sizes are achieved using a core/shell growth technique, although XRD analysis indicates that the larger sized particles have some strain which likely originates from crystal defects. The size augmentation can be enhanced using more reactive precursors, here lead hexyldecanoate, but there is some agglomeration due to the reagent's high reactivity.

Our survey of the literature returned few examples of using enhanced X-ray diffraction analyses techniques for characterizing semiconductor quantum dots. While the small sizes of most QD systems obliviates the need to do so, we demonstrate here that great care must be taken when characterizing larger particles (>10 nm). To summarize, we hope that colloidal techniques may be studied and refined further to prepare larger nanomaterials, which may have enhanced efficacy for electronic applications which will pave the way for future research opportunities.

## Author contributions

TA synthesized and analyzed samples and prepared the manuscript. AH assisted with characterizations of samples. PS assisted with data analysis and manuscript preparation.

### Conflict of interest statement

The authors declare that the research was conducted in the absence of any commercial or financial relationships that could be construed as a potential conflict of interest.
