# Supplementary material for: Colloidal Synthesis of Bulk-Bandgap Lead Selenide Nanocrystals
Source: Front Chem. 2018 Nov 22;6:562. doi: 10.3389/fchem.2018.00562 (PMC6261983; doi:10.3389/fchem.2018.00562)
Supplement: Supplementary file 1 [file Data_Sheet_1.docx]

Supplementary Material

Colloidal Synthesis of Bulk-Bandgap Lead Selenide Nanocrystals

Thulitha M. Abeywickrama, Asra Hassan, and Preston T. Snee*

*** Correspondence:** Preston T. Snee : sneep@uic.edu

| *Index* | *Page* |
| --- | --- |
| **Figure S1.** NMR characterization of lead hexyldecanoate, Pb(C_16_H_31_O_2_)_2_. | 2 |
| **Table S1**: Combustion analysis of lead hexyldecanoate, Pb(C_16_H_31_O_2_)_2_. | 2 |
| **Figure S2.** X-ray photoelectron spectroscopy spectrum of “core” PbSe QDs prepared by Method 2 using lead oleate. | 3 |
| **Figure S3.**  TEM images of “core” PbSe QDs prepared by Method 2 using lead oleate. | 3 |
| **Table S2**: XPS composition analysis of “core” PbSe QDs prepared by Method 2 using lead oleate. | 4 |
| **Figure S4.** TEM images of overcoated PbSe using lead oleate at 160 °C. | 4 |
| **Figure S5.** TEM images of overcoated PbSe using lead oleate at 190 °C. | 4 |
| **Figure S6.** TEM images of overcoated PbSe using excess lead oleate and selenium precursors at 160 °C. | 5 |
| **Figure S7.** TEM images of overcoated PbSe using lead hexyldecanoate at 160 °C. | 5 |
| **Figure S8.** TEM images of overcoated PbSe using lead hexyldecanoate at 130 °C. | 6 |
| Williamson-Hall analysis of XRD lineshapes. | 6 |
| **Figure S9.** Williamson-Hall analysis of PbSe | 6-7 |


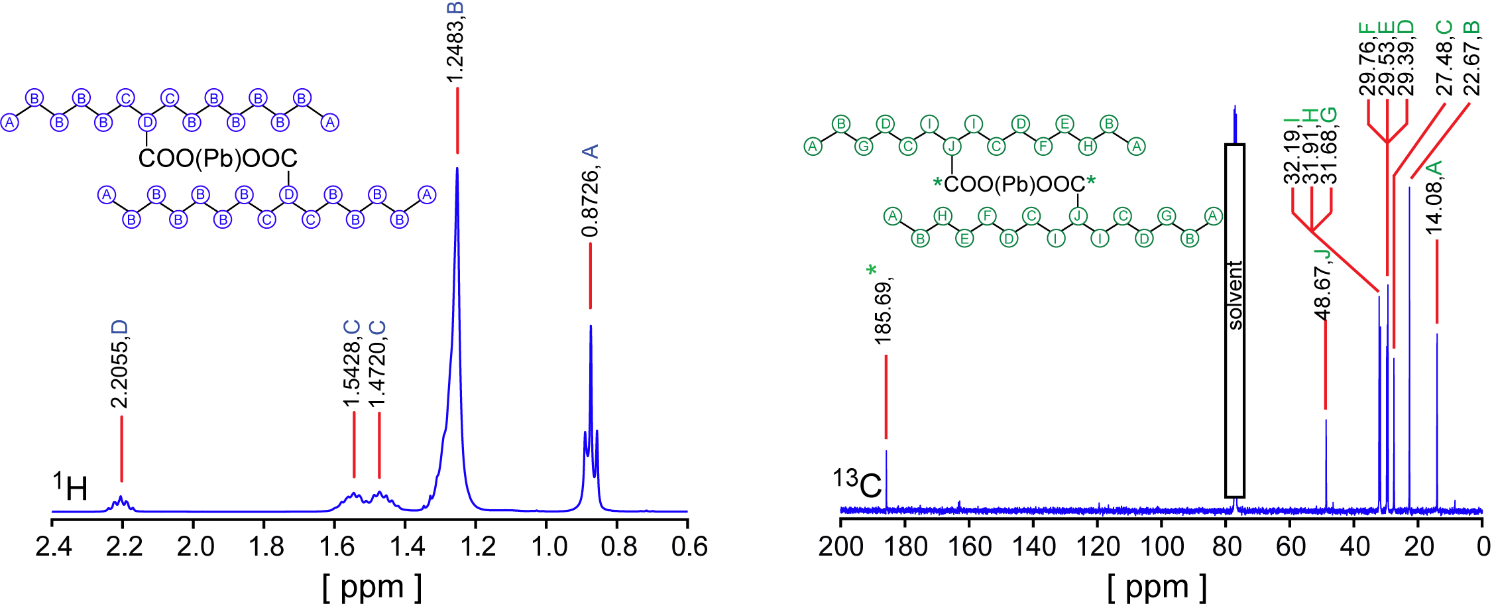


**Figure S1.** ^1^H (left) and ^13^C (right) NMR characterization of lead hexyldecanoate, Pb(C_16_H_31_O_2_)_2_. Relevant peak positions are indicated by letters.

**Table S1.** Elemental analysis for lead hexyldecanoate, Pb(C_16_H_31_O_2_)_2_. Deviations from the expected stoichiometry are due to residual triethylamine in the product.

| **Element** | **% found** | **% expected** |
| --- | --- | --- |
| C | 45.51 | 53.53 |
| H | 7.48 | 8.70 |

**
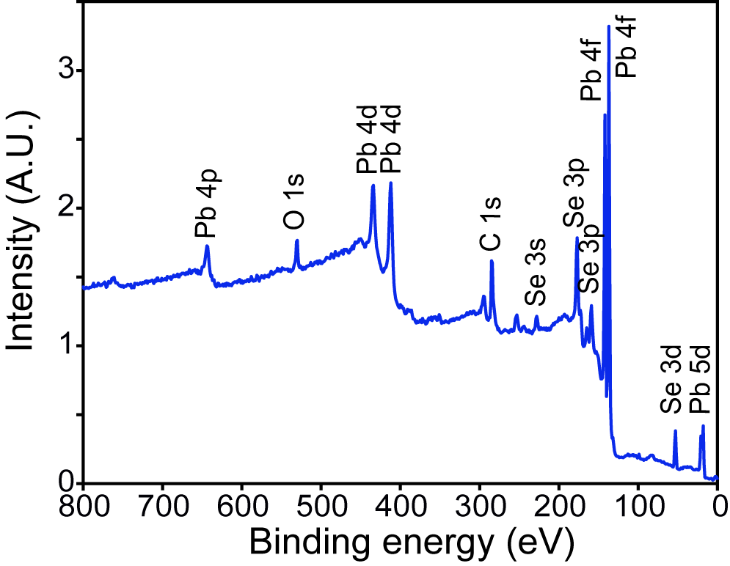
**

**Figure S2.** X-ray photoelectron spectroscopy (XPS) survey results of the “core” PbSe QDs formed by lead oleate precursor under the extra ligand condition (Method 2).

**
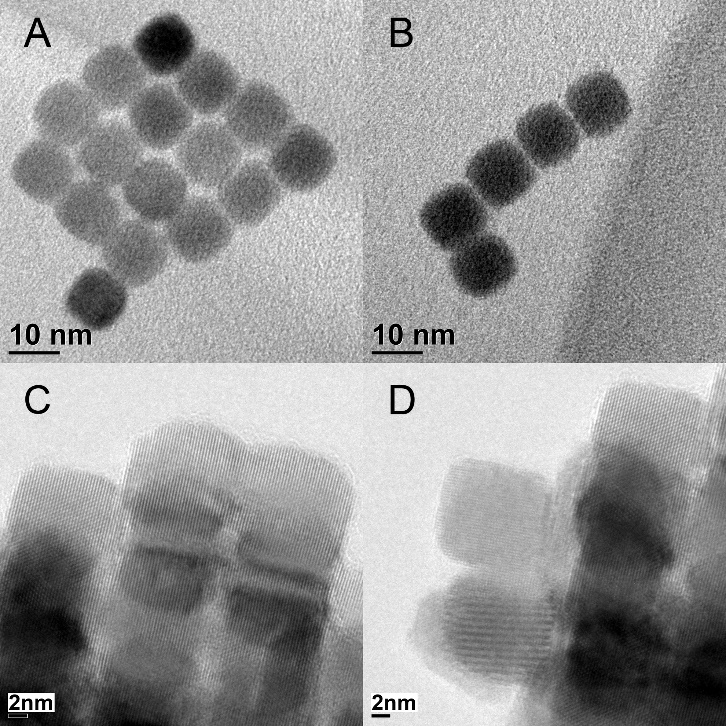
**

**Figure S3.** Low resolution (a,b) and high resolution (c,d) TEM micrographs of the PbSe QD cores prepared by Method 2 using a lead oleate precursor. Additional images and data are provided in Figure 2 C, D of the main text.

**Table S2**: XPS elemental analysis of “core” PbSe QDs prepared by Method 2 using lead oleate.

| ***Sample*** | ***Pb%*** | ***Se%*** | ***O %*** |
| --- | --- | --- | --- |
| PbSe | 42.96 | 24.04 | 32.99 |


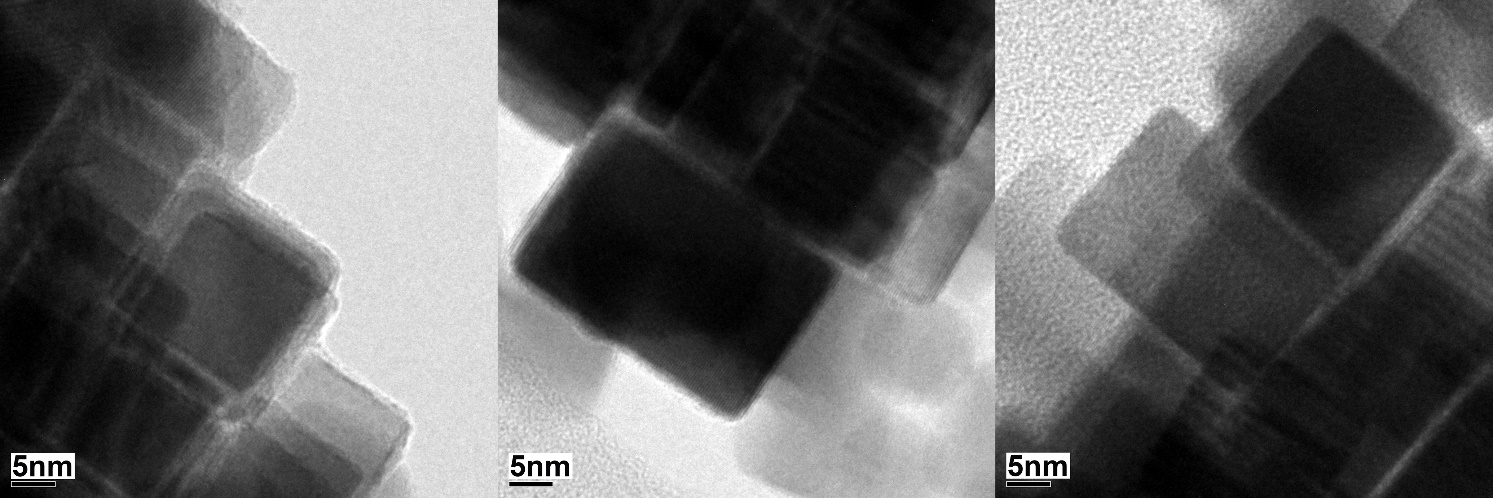


**Figure S4.** TEM micrographs of overcoated PbSe nanocrystals using the lead oleate precursor (overcoating Method 2) at 160 °C.


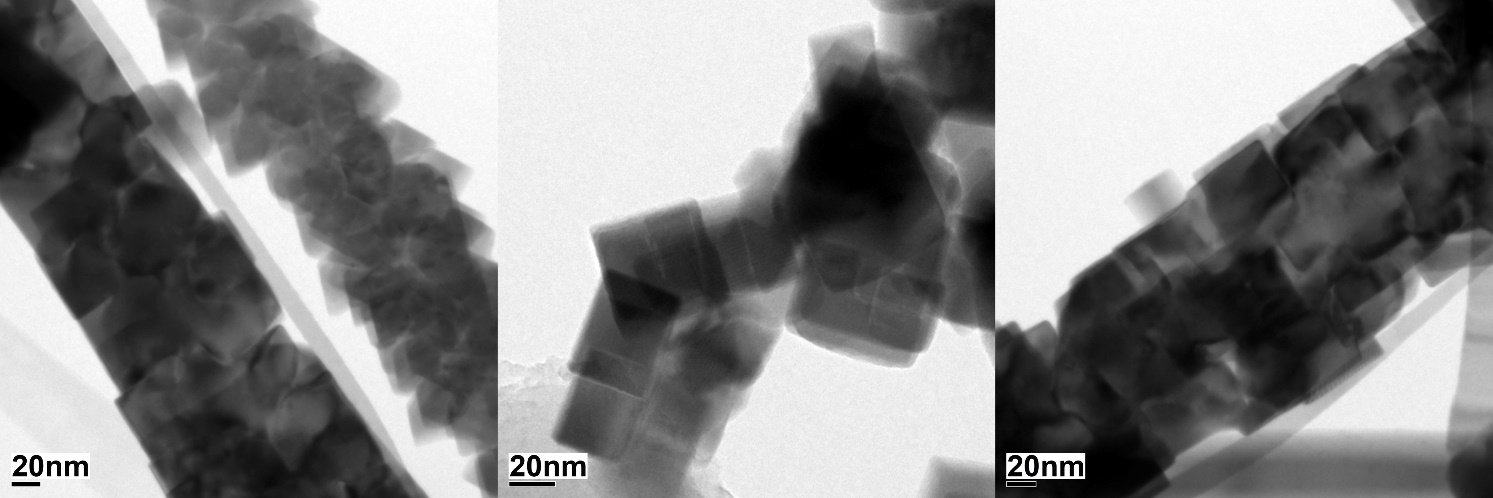


**Figure S5.** TEM micrographs of overcoated PbSe nanocrystals using the lead oleate precursor (overcoating Method 2) at 190 °C.


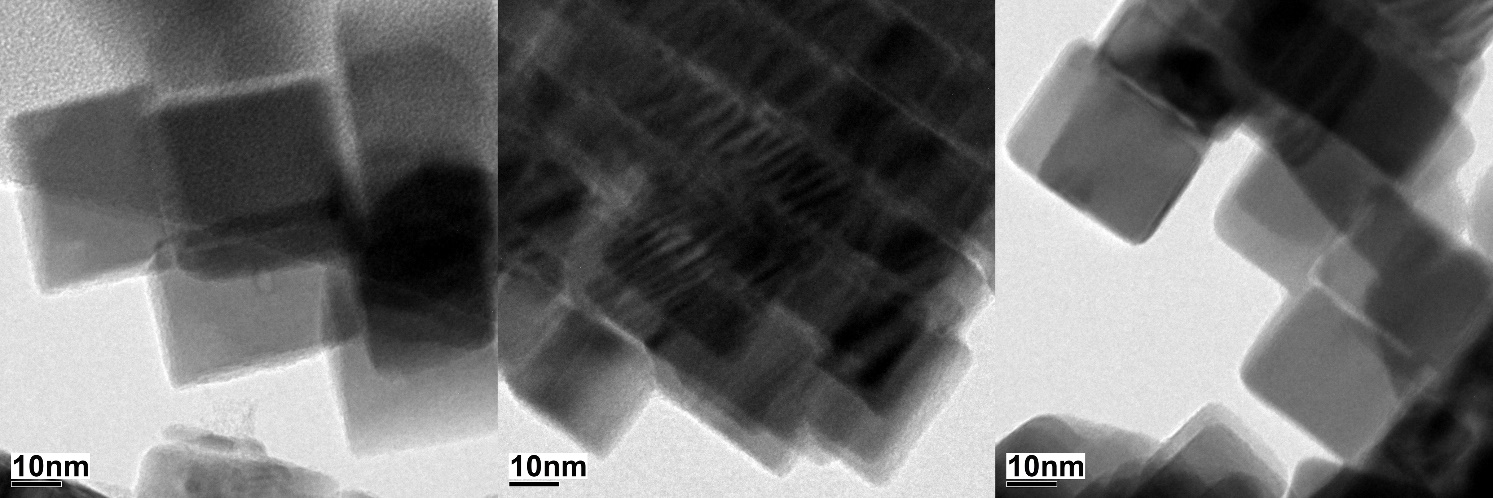


**Figure S6.** TEM micrographs of overcoated PbSe nanocrystals using overcoating Method 1 at 160 °C, whereby the shell precursors were raised by 2×.





**Figure S7.** TEM micrographs of overcoated PbSe nanocrystals using lead hexyldecanoate precursor (overcoating Method 2) at 160 °C.


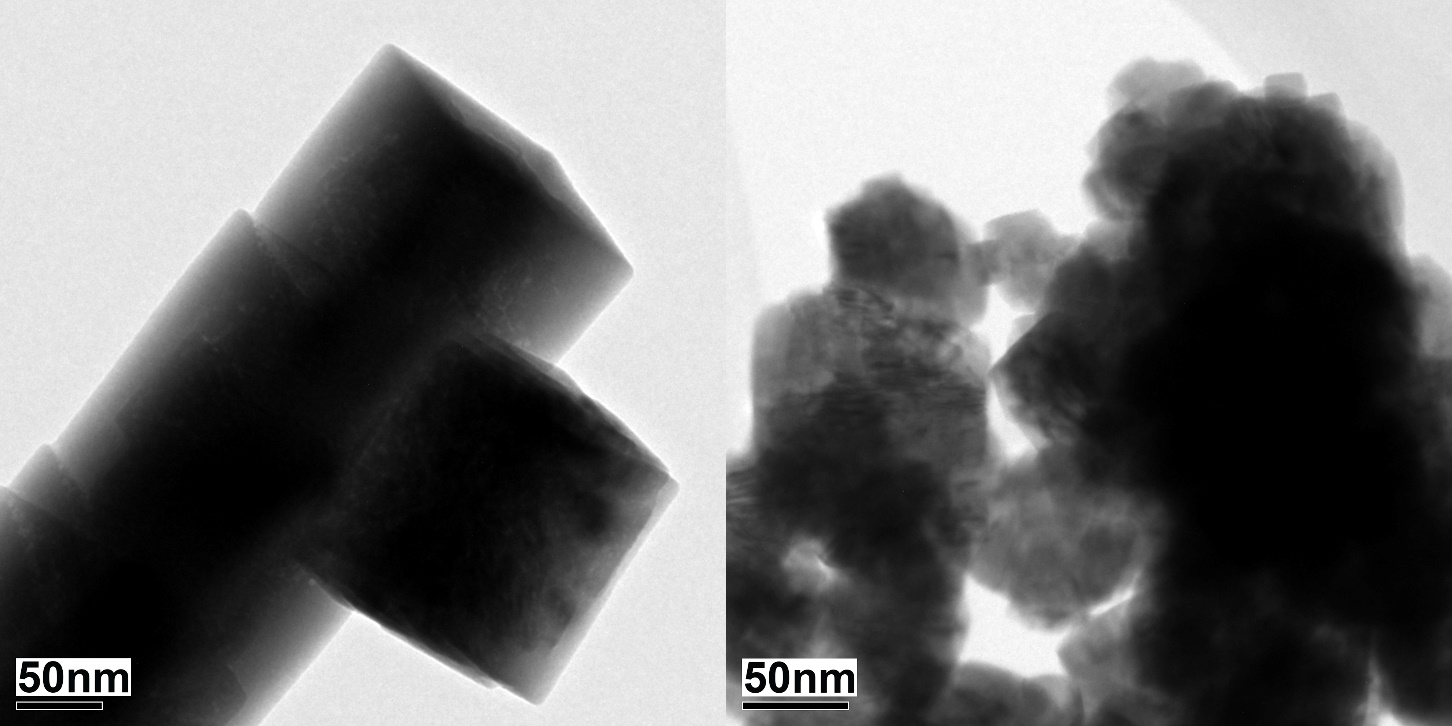


**Figure S8.** TEM micrographs of overcoated PbSe nanocrystals using lead hexyldecanoate precursor (overcoating Method 2) at 130 °C.

**Williamson-Hall Analysis**

XRD lineshape analyses was performed by generating a Voigt fit to a resonance, convolving it with the instrument response generated from the NIST standard, and using the result to calculate χ^2^ for optimizing the Voigt fit. Data were collected both at low resolution and high signal, and vice versa. The effect of the diffractometer’s resolution was minimal so long as the data were corrected for the instrument response as shown in Figure S9 C below.

**
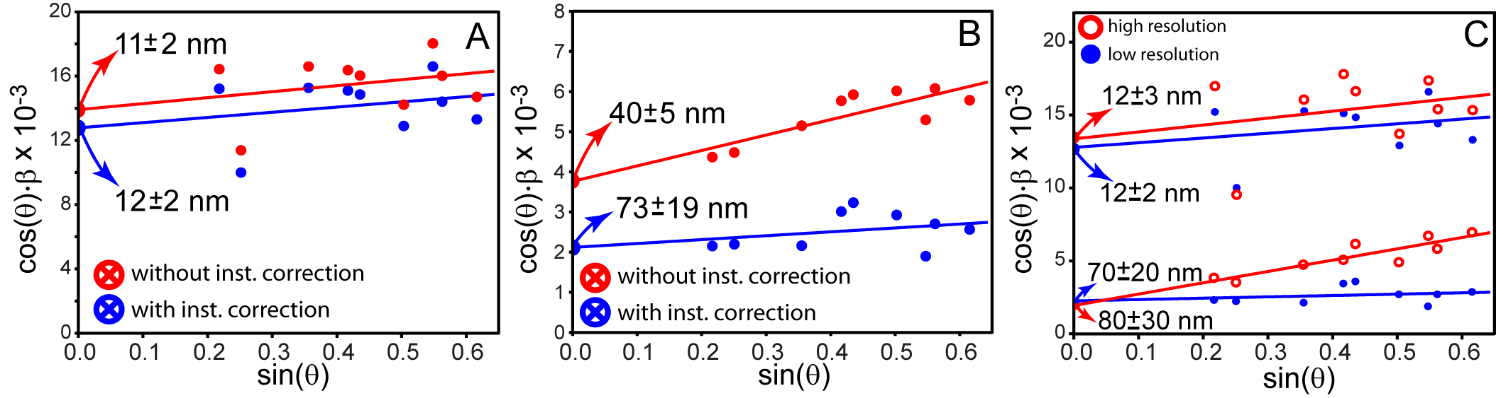
**

**Figure S9.** **A.** Williamson-Hall linear regressions of ~12 nm PbSe QDs and larger PbSe agglomerates (**B**) with (blue) and without (red) deconvolution of the instrument response function. The error due to a lack of adequate data processing on the characterization of small QDs is rather minimal. However, significant error is encountered when analyzing larger particles unless the XRD data have been processed to remove the effects of instrument broadening. **C**. The effects of high and low diffractometer instrument resolution are minimal when determining the sizes of particles using a Williamson-Hall regression, so long as a best attempt has been performed for deconvolving the instrumental broadening from the data. It is noticeable in this data set that the larger PbSe agglomerated nanocrystals appears to have less strain when analyzed at high resolution. This could be due to the fact that this sample is composed of “core” PbSe prepared using lead hexyldecanoate, and thus perhaps should not have the strain of overcoated PbSe.
